# A structural annotation resource for the selection of putative target proteins in the malaria parasite

**DOI:** 10.1186/1475-2875-7-90

**Published:** 2008-05-23

**Authors:** Yolandi Joubert, Fourie Joubert

**Affiliations:** 1 Bioinformatics and Computational Biology Unit, Department of Biochemistry, University of Pretoria, Pretoria, 0002, South Africa

## Abstract

**Background:**

Protein structure plays a pivotal role in elucidating mechanisms of parasite functioning and drug resistance. Moreover, protein structure aids the determination of protein function, which can together with the structure be used to identify novel drug targets in the parasite. However, various structural features in *Plasmodium falciparum *proteins complicate the experimental determination of protein structures. Limited similarity to proteins in the Protein Data Bank and the shortage of solved protein structures in the malaria parasite necessitate genome-scale structural annotation of *P. falciparum *proteins. Additionally, the annotation of a range of structural features facilitates the identification of suitable targets for experimental and computational studies.

**Methods:**

An integrated structural annotation system was developed and applied to *P. falciparum*, *Plasmodium vivax *and *Plasmodium yoelii*. The annotation included searches for sequence similarity, patterns and domains in addition to the following predictions: secondary structure, transmembrane helices, protein disorder, low complexity, coiled-coils and small molecule interactions. Subsequently, candidate proteins for further structural studies were identified based on the annotated structural features.

**Results:**

The annotation results are accessible through a web interface, enabling users to select groups of proteins which fulfil multiple criteria pertaining to structural and functional features [1]. Analysis of features in the *P. falciparum *proteome showed that protein-interacting proteins contained a higher percentage of predicted disordered residues than non-interacting proteins. Proteins interacting with 10 or more proteins have a disordered content concentrated in the range of 60–100%, while the disorder distribution for proteins having only one interacting partner, was more evenly spread.

**Conclusion:**

A series of *P. falciparum *protein targets for experimental structure determination, comparative modelling and *in silico *docking studies were putatively identified. The system is available for public use, where researchers may identify proteins by querying with multiple physico-chemical, sequence similarity and interaction features.

## Background

Malaria parasite resistance to therapeutic drugs such as sulfadoxine and pyrimethamine have increased significantly during the past two decades [2,3]. Following the rise in resistance, there has been a pressing need to understand the mechanism of drug resistance and develop novel anti-malarial drugs. Protein structure has previously been used to elucidate the mechanism of resistance in *Plasmodium falciparum *[4,5]. Furthermore, inhibitors can be designed from structure [6,7]. As resistance to existing drugs is a globally occurring phenomenon, new information regarding the structure and function of the proteins in especially the *P. falciparum *genome is of importance. However, various features of the parasite genome and proteome complicate functional and structural characterization studies, including a high AT-content and the presence of low complexity regions and inserts [8,9].

Structural and functional information is limited for *P. falciparum *proteins. To illustrate, a search of the PDB using "falciparum" as keyword retrieved 210 structures at the time of writing. Once sequences with more than 90% sequence identity were removed, 103 structures remained [10]. Regarding functional annotation, Gene Ontology terms [11] have been assigned manually to around 40% of all *P. falciparum *gene products [8]. Almost 60% percent of the proteins do not have sufficient similarity to known proteins and therefore no function can be assigned to them. In short, 4% of *P. falciparum *proteins have experimental three dimensional structures assigned, and 60% of the proteome is described as hypothetical. Furthermore, the amount of redundant *P. falciparum *proteins in the PDB is significant.

Generating experimental data to provide evidence of protein structure and function is expensive, difficult and slow. Conversely, predictive computational methods are fast and applicable to whole proteomes. Although they are less reliable than experimental results, predictions can identify proteins of interest and determine their suitability for experimental studies [12,13]. Moreover, knowledge of the structural features of proteins guides experiment design [14].

Methods used for three dimensional protein structure prediction are primarily based on homology transfer. Structure is more conserved than sequence and therefore distantly related sequences often have the same or very similar structures [15]. Computational methods for structure feature prediction make use of machine learning, statistical methods and physical properties of amino acid sequences. Typical computer-based methods for structural annotation include the prediction of secondary structure, transmembrane helices, low complexity, disorder, coiled-coils, and 3D structure.

Integrating these annotations are important for three major reasons: Different databases cover different sets of proteins; prediction methods have different strengths and weaknesses and finally, biological conclusions about function and structure can be derived more accurately considering as much information as possible about a certain sequence. Therefore, many meta-servers and integrated databases for genome-scale protein structural and functional annotation have been generated. Proteome annotation with regard to structure and function is important for comparative studies [16] and for selecting sets of proteins of particular interest from an organism [12].

This study entailed the development of an automated structural annotation pipeline for the malaria parasite [1] and the semi-automated annotation of additional features in the *P. falciparum*, *P, vivax *and *P. yoelii *genomes. In addition, the number of proteins with specific predicted features was calculated. Finally, lists of putative candidates for further experimental and *in silico *structural studies were compiled. It is not intended to compete with the established PlasmoDB database [17], but attempts to provide a supplementary specialized environment for performing complex queries based on structural and other properties, enabling researchers to select molecules with specific properties for further investigation. It does make use of information from, and provide links to the PlasmoDB site.

## Methods

Development was done in Python, utilizing the Zope web application framework with a PostgreSQL database. Protein sequences were obtained from PlasmoDB release 5 [17]. Data sources were the *Plasmodium falciparum *[8], *Plasmodium vivax *[18] and *Plasmodium yoelii *[19] genome sequencing projects. All annotated proteins were used. Analyses were performed on a 64× CPU Linux cluster. Protein statistics were gathered using Pepstats from EMBOSS [20]. BLAST searches were done using NCBI BLAST 2.2.10 [21] against the PDB [22] with a E-value cut-off value of 20. HMMPfam from the HMMER package [23] was run against the Superfamily database [24] with an e-value cut-off of 1e-^1 ^for protein structural family classification. Threading was done with Threader 3 [25], using secondary structure predictions from PsiPred [26]. Only sequences shorter than 400 residues were used. Transmembrane helix predictions were done using TMHMM2 [27]. The EMBOSS program, SigCleave was used to predict signal peptides, and Paircoil2 [28] was employed to predict coiled-coil regions. Secondary structure predictions were performed with three iterations of PsiPred 2.5. Protein disorder was predicted with the Disprot/VSL2 predictors [29]. SMID-BLAST 1.02 was used to analyse possible protein-ligand interactions (Unleashed Informatics), no e-value cutoffs were implemented. Motifs were analysed with pscan and patmatmotifs from EMBOSS. For protein-protein interactions, data from high-throughput yeast-two hybrid experiments [30] were annotated to the malaria sequences. Proteins previously predicted to be exported out of the red blood cell [31,32] were annotated.

For the identification of candidate proteins for homology modelling, sequence similarity with a protein in the PDB was required. Contrastingly, candidate selection for X-ray crystallization required that proteins did not have sequence similarity with proteins in the PDB. Protein sequences with more than 30% predicted coiled-coils, disorder, transmembrane regions and signal peptides were eliminated. The SMID-BLAST predictions were used to identify proteins to which small molecules bind and which might be suitable for *in silico *docking studies. In addition, these proteins had to have a crystal structure or good sequence similarity in the PDB.

## Results and Discussion

### The structural annotation system

**Figure 1:**
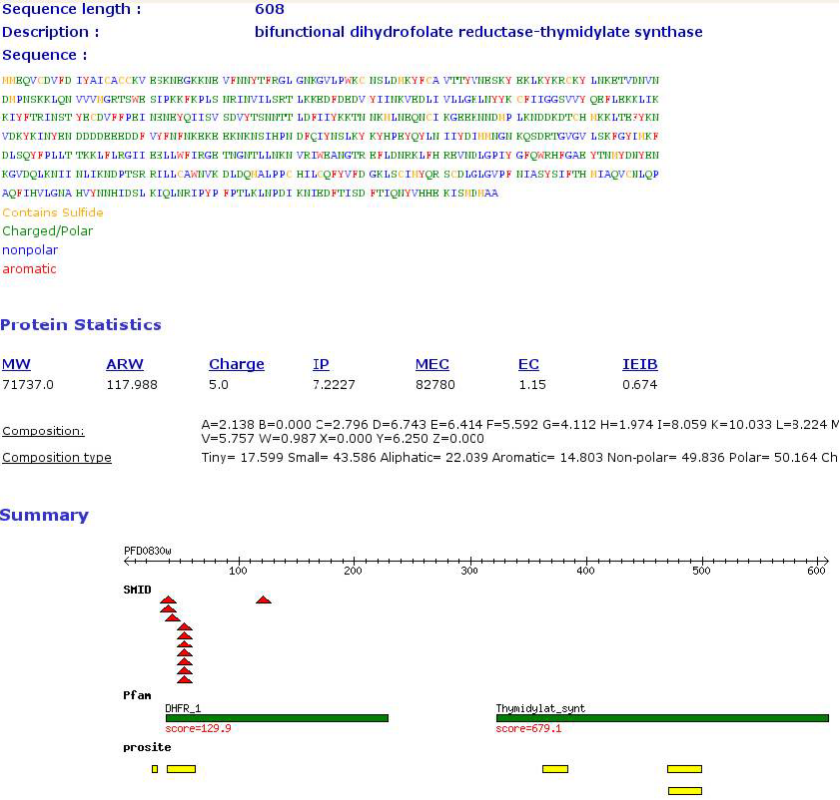
**An example of a part of the protein information view for the *P. falciparum *protein, bifunctional dihydrofolate-reductase/thymidylate synthase.** The sequence is colored by physical-chemical properties. Protein statistics shown include molecular weight (MW), average residue weight (ARW), charge, iso-electric point (IP), molar extinction coefficient (MEC), extinction coefficient at 1 mg/ml (EC) and improbability of expression in inclusion bodies (IEIB). SMID (red triangles), Pfam (green bars) and Prosite (yellow bars) hits are graphically indicated along the length of the protein.

Using the web interface, proteins can be searched by keywords, by browsing per chromosome and by designing complex inclusion and exclusion queries using an intuitive check-box and form interface. Following selection and filtering, an individual protein's result page starts with sequence, followed by statistics as calculated by pepstats. The next section provides the user with a summary image displaying database coverage, motifs, disordered regions, coiled-coils, low complexity and transmembrane helices (Figure 1).

Each of the subsequent sections lists the results of a specific analysis. The results include start and end positions on the query sequence, scores, e-values, links to other databases and descriptions. A graph constructed by Matplotlib then displays the confidence values for helix, strand and disorder predictions over the length of the protein sequence. BLAST PDB hits as well as Threader results are displayed in a tabular format, with links to the relevant protein structures. Similarly, protein-protein and small molecule interactions are reported in tabular form, together with the relevant links. Pfam domains and Superfamily results are represented graphically together with links to the relevant entries. Subsequently, patterns and disordered regions are graphically displayed. Metabolic pathway information is summarized, and sequence similarity between *Plasmodium *species is presented.

### Analysis of the *P. falciparum *proteome

A short summary of the results discussed here is provided in Table 1. Twenty-seven percent of proteins had BLAST/PDB hits with at least 25% identity to the hit. One third of these proteins (10% of the proteome) had at least two-thirds of their sequence covered by a PDB match. Almost 20% of the sequences had at least one-third of the length covered by a PDB hit. An additional 413 sequences had Superfamily hits with a score of 100 or better. Therefore, an estimate of proteins which could be assigned to an existing fold is 1 224 or 23%. Finally, out of 2,462 proteins subjected to threading, 423 had alignments with Z-scores better than 3.95. Out of these, about 100 did not have BLAST-PDB matches with e-values smaller than 0.5, which covered more than 30% of the query sequence.

**Table 1:** A summary of selected features calculated for annotated proteins in *Plasmodium falciparum *(a total of 5,411 proteins were analysed).

**Feature**	**Occurrence**
BLAST vs. PDB hits with at least 25% identity	27%
Hits vs. Pfam with E-value < 1 × 10^-15^	32%
No Pfam hits	43%
Threading Z score > 3.95	8%
One or more predicted coiled-coil regions	10%
Predicted to be transported out of the RBC (Pexel)	5%
Predicted to contain at least one transmembrane helix	30%
Predicted small molecule binding by SMID	22%
Protein-protein interaction by yeast-two hybrid results	15%
Predicted ≤ 40% disorder or no regular secondary structure	60%
Mean percentage low complexity per sequence	16%

Thirty-two percent of proteins had hits in Pfam below an e-value of 1 × 10^-15^. At least 43% of all sequences had no hits with families in Pfam. There were 1,987 sequences with hits in Pfam with an e-value smaller than 1 × 10^-10^. An additional 332 sequences had PRINTS hits. Thus, a total of 2 319 sequences or 43% of the annotated proteome could be assigned to functional families making use of the Pfam and PRINTS databases. Almost 200 proteins had Superfamily hits with an e-value smaller than 1 × 10^-3^. Of these, 650 sequences did not have Pfam or PRINTS matches.

Ten percent of proteins had one or more predicted coiled-coil region, 5% are predicted to be transported out of the red blood cell based on the presence of the Pexel motifs, and about 30% of proteins were predicted to contain at least one transmembrane helix. At least 22% of proteins were predicted to bind to small molecules by SMID-BLAST. Almost 15% of the proteins interact with other proteins according to the high-throughput yeast-two hybrid experiments. Sixty percent of the proteins were predicted to contain at least 40% intrinsic disorder or no regular secondary structure.

As with other genomes, the most abundant transmembrane proteins contain only one transmembrane helix [33]. The amount of transmembrane proteins decrease as the amount of membrane spanning helices increase, with the exception of 6-tm and 11-tm proteins which are slightly more than the portion of 5-tm and 10-tm proteins, respectively. The correlation between intrinsic disorder and interacting proteins was investigated. The mean percentage disorder in interacting sequences is 61%, while the mean percentage disorder in non-interacting proteins is 44% and the overall mean percentage disorder for all sequences is 48%. In agreement with previous studies of interacting proteins in human and their disorder content, *P. falciparum *interacting proteins contain higher intrinsic disorder content than non-interacting proteins. Because disorder in a protein makes it more flexible, it was expected that the disorder content would increase with the number of interacting partners. For proteins interacting with only one other protein, the predicted disorder varies from 4% to 100%. The majority of interacting proteins interact with less than 10 other proteins. As the amount of proteins decreases with an increasing number of interacting partners, the range of variation in disorder in the proteins also decreases, as expected. The ranges tend to span higher percentages of disorder as the amount of interacting partners increase.

### Inter-species comparisons

The proteins from the *P. falciparum *length distribution have a longer tail than the other two species, and *P. yoelii *has a more symmetrical length distribution than the other species. The mean length for *P. vivax *is 630 with a standard deviation of 576 amino acids and the mean length for *P. yoelii *is 420 with a standard deviation of 450. The proteins in *P. yoelii *vary less in length than in the other two species, with *P. falciparum *showing the most variation. Asparagine is the most abundant amino acid in *P. falciparum *and *P. yoelii*, and Lysine in *P. vivax*. Although lysine is the most abundant amino acid in *P. vivax*, it should be noted that lysine is less abundant in *P. vivax *(9%) than in the other two species (11.5%). *Plasmodium vivax *contains on average twice as many alanine and glycine as the other two species. Overall, 26% of residues in *P. vivax *are tiny (A, C, G, S, T), in comparison to the 18% and 19% tiny residues contained within *P. falciparum *and *P. yoelii*, respectively.

*Plasmodium vivax *contains more proteins with small percentages of low complexity. Although *P. yoelii *and *P. falciparum *contain the same amount of proteins with predicted low complexity regions, the proportion of *P. yoelii *proteins is much lower than for *P. falciparum*. *P. yoelii *and *P. falciparum *have similar proportions of disorder and order-promoting amino acids, whereas *P. vivax *has proportionally more disorder-favouring amino acids and less order-promoting amino acids. The average percentage low complexity per sequence is 16% in *P. falciparum*, 10% in *P. vivax*, and 12% in *P. yoelii*. No low complexity is predicted for 27%, 19% and 13% percent of the sequences in *P. yoelii*, *P. vivax *and *P. falciparum*, respectively. *P. yoelii *and *P. falciparum *have an equal portion of transmembrane proteins, while *P. vivax *has less predicted transmembrane proteins. *P. falciparum *has more 2-tm, 3-tm, 4-tm, 6-tm and 9-tm proteins than the other two species. *P. vivax *has slightly more 8-tm proteins than *P. yoelii *and *P. falciparum *and *P. yoelii *has the most 1-tm proteins.

### Identification of potential molecules for further study

Tables containing putative candidates possibly suitable for homology modelling can be viewed through the web interface [34]. These proteins contain PDB matches with e-values better than 1 × 10^-20 ^and which have more than 70% of their sequence covered by the PDB match. The cut-off sequence identity was set to 25%. Therefore, these tables contain proteins for which high quality models could possibly be obtained through automatic model building. Separate tables contain interacting proteins, proteins with Pfam domains and uncharacterized proteins [35].

Proteins possibly suitable for *in silico *docking studies can also be accessed through the web interface [35]. These proteins were selected based on the presence of predicted small molecule binding sites and the availability of a 3D structure. Interacting proteins were separated from non-interacting proteins.

Possible targets for experimental structure determination are available for proteins with a Pfam domain [36] and for proteins without a Pfam domain [37]. A lack of significant BLAST hits to entries in the PDB formed part of the basis for the putative identification of possible new targets for X-ray crystallography. For these, priority categories were determined, which are explained in Table 2.

**Table 2:** The number of targets suggested for further study using experimental structural elucidation techniques in each priority category (PC, ranked 1 – 6) after the relevant elimination step.

**Priority Class (PC)**	**PDB E-value range**	**Nr of proteins**	**Tm + disorder**	**CC+LC+SP**	**a**	**b**
PC1	No PDB matches	139	11	8	1	7
PC2	E-value > 10	174	15	11	3	8
PC3	10 >= E-value > 5	352	34	31	9	22
PC4	5 >= E-value > 3	332	35	19	5	14
PC5	3 >= E-value > 1	810	88	51	12	39
PC6	1 >= E-value > 0.5	529	60	58	10	48

Tm+disorder refers to the amount of proteins in each group after the transmembrane and disorder filtering step. CC+LC+SP refers to the proteins in each group after coiled-coils, low complexity and signal peptide filtering step. Group a indicates proteins containing a Pfam functional domain. Group b indicates proteins without a Pfam functional domain.

## Conclusion

In order to allow researchers to select groups of proteins which fulfil certain criteria with regard to structural and functional features, a semi-automated structural annotation of selected species of the malaria parasite as performed, and a web-based resource with query functionality was developed. This tool was used to gather statistics regarding a series of structural and functional characteristics. Furthermore, a series of putative candidate proteins for homology modelling, crystallization and docking studies were generated.

It is important to realize that the results presented her are dependent on the genome data and gene predictions available at the time of analysis. In the case of *Plasmodium *falciparum, a recent article has highlighted the shortcomings in the current state of gene prediction for malaria parasites, based on cDNA analysis [38]. Furthermore, this study is based on *P. falciparum *data from PlasmoDB 5.0, and a draft re-annotation of this genome has recently taken place. It is planned to incorporate the relevant results as soon as possible, Also, the *P. vivax *data should be regarded as preliminary as the genome is still unfinished, with a publication expected soon [39]. It is hoped that this web-based resource may be valuable for researchers aiming to identify malaria proteins with specific combinations of sequence, structural and interaction features for further studies.

## Authors' contributions

FJ conceived the project, obtained funding and supervised the study. YJ performed the software, database and interface development, investigated the occurrence of the different features described, performed the inter-species comparison and compiled the lists of possible targets for further studies. Both authors prepared the manuscript.
